# A Label-Free Aptasensor for the Detection of Sulfaquinoxaline Using AuNPs and Aptamer in Water Environment

**DOI:** 10.3390/bios15010030

**Published:** 2025-01-08

**Authors:** Zhaoyang Zhou, Xingyue Chen, Shuang Jiang, Zhuoer Chen, Sixian Wang, Yueyang Ren, Xiaodong Fan, Tao Le

**Affiliations:** Chongqing Key Laboratory of Conservation and Utilization of Freshwater Fishes, Animal Biology Key Laboratory of Chongqing Education Commission of China, College of Life Sciences, Chongqing Normal University, Chongqing 401331, China

**Keywords:** sulfaquinoxaline, AuNPs, aptasensor, residue, environment

## Abstract

Sulfaquinoxaline (SQX) is widely utilized in aquaculture and animal husbandry due to its broad antimicrobial spectrum and low cost. However, it is difficult to degrade, and there are relevant residues in the aquatic environment, which could be harmful to both the ecological environment and human health. As a new recognition molecule, the aptamer can be recognized with SQX with high affinity and specificity, and the aptamer is no longer adsorbed to AuNPs after binding to SQX, which weakens the catalytic effect of AuNPs. Consequently, an aptasensor for the detection of SQX was successfully developed. This aptasensor exhibits a linear range of 40–640 ng/mL, with a detection limit of 36.95 ng/mL, demonstrating both sensitivity and selectivity. The recoveries of this aptasensor in water samples ranged from 90 to 109.9%, which was quite in line with high-performance liquid chromatography. These findings suggest that the aptasensor is a valuable tool for detecting SQX in aqueous environmental samples.

## 1. Introduction

Sulfaquinoxaline (SQX) is a commonly used sulfonamide veterinary antibiotic with the advantages of broad antimicrobial spectrum and low price, and is widely used for treating and preventing bacterial infections in aquaculture and animal husbandry [1,2]. However, SQX degrades slowly and persists in the environment, with residual SQX detectable in both surface water and groundwater [3]. SQX in a water environment can enter the food chain through direct consumption or by aquatic organisms and crops, which may cause allergies, affect intestinal bacteria, lead to digestive and urinary dysfunction, and jeopardize human health [4,5,6]. Consequently, the maximum residual limit for SQX has been established at 100 μg/kg in several countries, including China, the United States, and European nations [7,8]. Therefore, it is critical to develop an effective and rapid assay that can detect SQX residues.

There are currently several detection techniques used to detect SQX residues. The more common methods are high-performance liquid chromatography (HPLC) [9,10,11], liquid chromatography-tandem mass spectrometry (LC-MS/MS) [12], ultra-high performance liquid chromatography-mass spectrometry (UHPLC-MS/MS) [13,14], and capillary electrophoresis (CE) [15,16], which have high sensitivity and stability, but the equipment is expensive, complicated to operate, and requires specialized personnel. In addition, immunoassay methods for detecting of SQX, such as immunochromatographic assays (ICA) [17], have been reported to provide rapid and highly sensitive results. However, it is difficult to prepare antibodies that recognize SQX, and the antibodies vary widely from batch to batch. Therefore, a novel and economical molecular probe for recognizing SQX is urgently needed. Aptamers, first proposed by Ellington [18] and Tuerk [19], are a kind of single-stranded DNA or RNA molecule selected from a random nucleic acid sequence library using SELEX technology that have the advantages of a shorter fabrication period, lower production cost, and better stability compared with antibodies [20,21]. Aptamers usually fold into some specific structures and recognize the target with high affinity and specificity through hydrogen bonds, van der Waals interactions, and other mechanisms [22,23].

As a kind of functional oligonucleotide, aptamers can be applied in various fields, including biosensing, medical imaging, and targeted drug delivery [24,25,26]. Currently, the preparation of various aptasensors using aptamer-conjugated nanomaterials has become a research hotspot for environmental monitoring and food safety hazard detection. Zheng et al. [27] constructed a fluorescent aptasensor based on graphene oxide, where one end of the aptamer that specifically binds to 1-aminolevulinic acid is modified with a FAM-labeled fluorescence, and a high-specificity and high-sensitivity detection of the target can be achieved by generating fluorescence resonance energy transfer. Tang et al. [28] developed a dual fluorescent aptasensor based on silica composites using two aptamers that were modified by FAM and CY5 and immobilized on a silica surface by a carboxyl-modified cDNA linker, which demonstrated high sensitivity and specificity for sulfadimethoxine and oxytetracycline. Zhang et al. [29] labeled the 3′ end of Sul-01 with FAM as a fluorescence donor, and the 5′ end was labeled with NED as a fluorescence acceptor, as well as designed the quenching probe BHQ2-P_Q_-01 based on the fluorescence resonance energy transfer between FAM and NED to develop a fluorescence aptasensor for detecting SQX. Li et al. [30] immobilized double-stranded DNA on AuPd NPs@UiO-66-NH2/CoSe2 nanocomposites, and in the presence of SQX, its corresponding aptamer was released from the double-stranded structure and the electrical signals were increased accordingly, and thus, an electrochemical aptasensor for detecting SQX was designed. Most of these aptasensors for detecting SQX require a labeled modification of the aptamer, which may have some effect on the affinity of the aptamer and is not conducive to high-affinity binding between the aptamer and the target. Avoiding the use of expensive labels (e.g., fluorescent dyes and nanoparticles) to modify the aptamer would be more conducive to the binding between the aptamer and the target, and the aptasensor would be easier to prepare and use, making a label-free aptasensor cost-effective and suitable for widespread application.

Gold nanoparticles (AuNPs) are widely recognized as nanomaterials with potential applications. It has been reported that AuNPs can be used in aptasensors for the detection of targets by colorimetric methods in the past due to their advantages of simple synthesis, large specific surface area, and high chemical stability [31,32,33]. Yang et al. [34] constructed an aptasensor based on aflatoxin B1 chimeric ligands and AuNPs and utilized the peroxidase activity of AuNPs for the colorimetric detection of the corn oil of AFB1, and the aptasensor had high sensitivity and specificity, which is suitable for applications in food safety monitoring. Wang et al. [35] developed a noncompetitive colorimetric aptasensor for the sensitive detection of diarrheal shellfish toxins using aptamer and hybridization chain reaction-assisted AuNPs. The aptasensor showed high sensitivity and specificity for target toxins in shellfish samples. AuNPs have peroxidase-like activity, which can oxidize TMB and generate colorimetric signals in the presence of H_2_O_2_. Aptamers with surfaces that are negatively charged due to the dissociation of phosphate groups can bind to AuNPs, thus creating an electrostatic interaction with TMB and increasing their local concentration, resulting in more TMB oxidation and a stronger colorimetric signal. Given that aptasensors constructed with AuNPs are simple, cost-effective, and colorimetric signal can be detected [36,37], we developed a label-free aptasensor based on aptamer-functionalized AuNPs for colorimetric detection of SQX.

In this study, a label-free aptasensor for the rapid detection of SQX was developed using AuNPs and SQX aptamers screened by our laboratory. The aptasensor realized the rapid detection of SQX in different water samples, and the performance was compared with HPLC. The aptasensor is characterized by high specificity, low cost, and simple operation, which provides a novel method for the detection of SQX residues in water environments.

## 2. Materials and Methods

### 2.1. Materials and Reagents

The SQX-specific aptamer SBA28-1 (5′-CCCTAGGGG-3′) used in this experiment was screened by our laboratory [38,39] and synthesized by Sangon Biotech (Shanghai, China). SQX, sulfadimethoxine (SMM), sulfadimethoxine (SME), sulfadimethoxine (SDM), and sulfamethoxymethoxine (SMZ) were purchased from Sigma-Aldrich (St. Louis, MO, USA). Ofloxacin (OFL), oxytetracycline (OTC), chloramphenicol (CAP), and chlortetracycline (CTC) were purchased from Aladdin Biotechnology Inc. (Shanghai, China). AuNPs were synthesized following a previously established protocol [40].

### 2.2. Colorimetric Aptasensor Detection of SQX

Initially, 25 nM of the aptamers was incubated with a series of different SQX solution concentrations for 30 min at room temperature in an incubator with constant temperature and shaking. Following this incubation, 50 μL of AuNP solution was added, and after thorough mixing and shaking for 30 min, 1.2 mM of TMB and 1.2 M of H_2_O_2_ were added, and shaking was continued for an additional 10 min. The final volume of the reaction system was 300 μL. The absorbance value of the mixture was measured at 650 nm, and the ΔA_650_ value was calculated at different SQX concentrations (ΔA_650_ = A_0_ − A_SQX_, where A_0_ is the absorbance value without the addition of SQX solution, and A_SQX_ is the absorbance value after the addition of SQX solution). The aptamers were dissolved in binding buffer, while TMB and H_2_O_2_ were dissolved in sodium acetate buffer (pH 4.0).

### 2.3. Optimization of Conditions

First, different concentrations of aptamer (10, 15, 20, 25, and 30 nM) were incubated with 1 μg/mL of SQX for 30 min, and then 50 μL of AuNPs were added, mixed, and shaken for another 30 min. Then, 1.2 mM TMB and 1.2 M H_2_O_2_ were added, and the mixture was allowed to react for 10 min. The absorbance of the solution was measured, and the ΔA_650_ value was calculated by determining the difference between the absorbance of the blank group without added SQX (A₀) and that of the experimental group with SQX (A_SQX_) (ΔA_650_ = A₀ − A_SQX_).

In addition, to investigate the effect of pH on the experimental results, the pH of the solution was adjusted with glacial acetic acid to 3.0, 3.5, 4.0, 4.5, and 5.0. A 25 nM aptamer was then incubated with 1 μg/mL of SQX for 30 min. Following this, 50 μL of AuNPs were added and shaken for 30 min. Then, 1.2 mM TMB and 1.2 M H_2_O_2_ were added for a 10 min reaction, after which the absorbance values were measured and ΔA_650_ was calculated.

Next, to optimize the concentration of TMB, a 25 nM aptamer was incubated with 1 μg/mL of SQX for 30 min and then mixed with AuNPs for another 30 min. Different concentrations of TMB (0.8, 1.0, 1.2, 1.4, and 1.6 mM), along with 1.2 M H_2_O_2_, were added and reacted for 10 min, and then the absorbance of the solution was measured and the ΔA_650_ was calculated.

Finally, to optimize the concentration of H_2_O_2_, a 25 nM aptamer was incubated with 1 μg/mL of SQX for 30 min before adding AuNPs to react for another 30 min. Then, 1.2 mM TMB and different concentrations of H_2_O_2_ (0.3, 0.6, 0.9, 1.2, and 1.5 M) were added and reacted for 10 min. The absorbance of the solution was measured, and the ΔA_650_ was calculated to determine the optimal concentration of H_2_O_2_.

### 2.4. Analytical Performance of the Aptasensor

Next, we evaluated the performance of the aptasensor. To verify its sensitivity, various concentrations of SQX (20, 40, 80, 160, 320, 640, 1280, and 2000 ng/mL) were incubated with 25 nM of aptamer for 30 min and then added to AuNPs to continue the reaction for another 30 min. Next, 1.2 mM TMB and 1.2 M H_2_O_2_ were introduced, and the mixture was allowed to react for 10 min. The absorbance values of the solutions were measured, and the magnitude of ΔA_650_ was calculated. The standard curves were established at different concentrations of SQX, the limit of detection (LOD) was calculated as 3 SD/slope, where SD is the standard deviation of the blank sample and slope is the slope of the linear regression curve. On the other hand, various interfering substances including SMM, SMZ, SME, SDM, CAP, OFL, OTC, and CTC were chosen to replace SQX; the concentration of each antibiotic was 1 µg/mL. The 25 nM aptamer was incubated with these antibiotics for 30 min before being mixed with AuNPs for another 30 min. Then, 1.2 mM TMB and 1.2 M H_2_O_2_ were added and allowed to react for 10 min. The absorbance values at 650 nm were measured for both experimental and control groups, and the specificity of the aptasensor was determined by comparing the magnitude of ΔA_650_ produced by SQX with that of the different interferents.

### 2.5. Analysis of Real Samples

The aptasensor was evaluated using lake water and tap water to confirm the effectiveness and accuracy of the aptasensor. The samples were centrifuged to remove impurities with a 0.22 μm filter membrane, and the absence of SQX in the water samples was confirmed using HPLC. Then, 10 mL water samples were spiked with various concentrations of SQX (50, 100, and 150 ng/mL) for the spiked recovery test. Firstly, the SQX-containing water samples were incubated with the aptamer for 30 min, followed by the addition of 50 μL of AuNPs for another 30 min. Then, 1.2 mM TMB and 1.2 M H_2_O_2_ were added, and the mixture was allowed to react for 10 min before the absorbance values were measured. In addition, the same lake water and tap water containing different concentrations of SQX were analyzed by HPLC. The chromatographic column was a ZORBAX SB-C18 column (4.6 × 150 mm, 5 μm), and the mobile phases were 2% glacial acetic acid (A) and 100% methanol (B). Recovery was calculated using the following formula: (measured concentration/known concentration) × 100%. Each spiked sample was analyzed five times to ensure statistical reliability [41].

## 3. Results and Discussion

### 3.1. Principles of the Aptasensor

**Figure 1:**
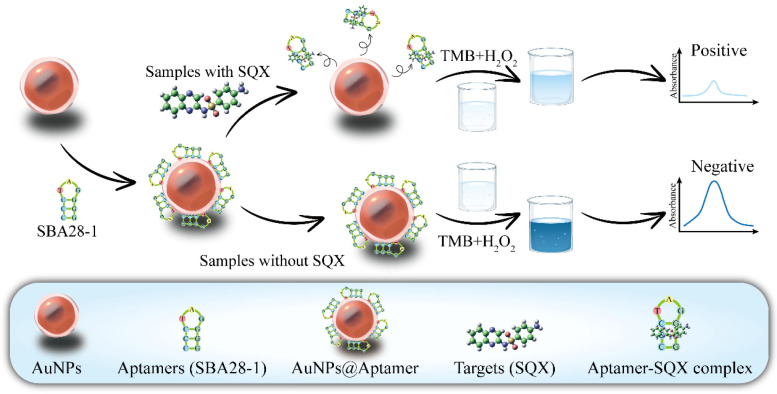
The principle of the aptasensor for detection of SQX.

The principle of SQX detection based on aptamers and AuNPs is illustrated in Figure 1. H_2_O_2_ is a common oxidant that oxidizes colorless TMB to blue TMB oxide, exhibiting an absorption peak at 650 nm [42]. In addition, AuNPs have peroxidase-like activity, which can be used as a catalyst to accelerate the process of this reaction. When the DNA aptamer binds to the surface of the AuNPs, its negatively charged phosphate backbone is exposed on the surface of the AuNPs. This increases the negative charge density of the aptamer–AuNP complex, enhancing its interaction with the positively charged substrate TMB and significantly boosting enzyme activity, resulting in the generation of more blue TMB oxides [43,44]. When SQX is added to the system, the aptamer binds to SQX and is no longer adsorbed onto the AuNPs, which weakens the catalytic effect of AuNPs, so that the TMB oxides produced in the same time are reduced and the solution changes from dark blue to light blue. Therefore, the concentration of SQX can be detected by measuring the absorbance value of the solution at 650 nm before and after the addition of SQX.

### 3.2. Feasibility of the Aptasensor

**Figure 2:**
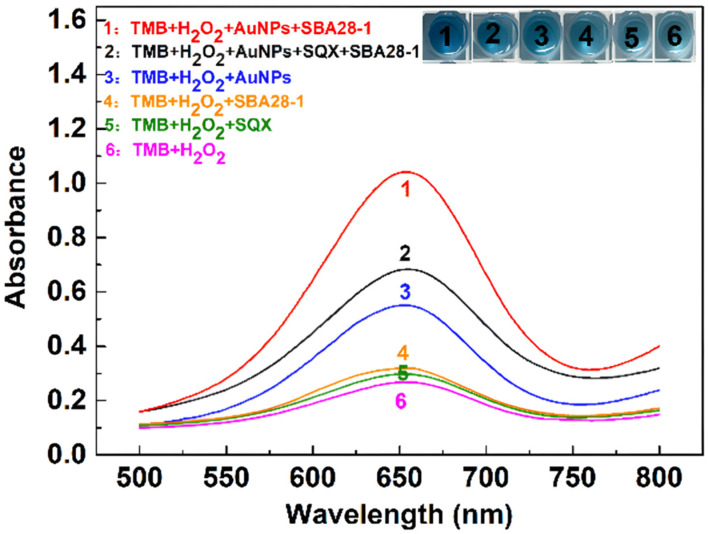
Absorption spectra of different solutions.

Different substances were added to the system of TMB and H_2_O_2_ for the study. As shown in Figure 2, curves 1 and 2 indicate that after the addition of SQX, the aptamer binds to SQX, the catalytic ability of AuNPs decreases, and the absorbance value of the solution at 650 nm decreases. Curves 1 and 3 show that the catalytic ability of AuNPs increased after the addition of the aptamer to the solution containing AuNPs, and the blue color of the solution became darker. According to curves 3 and 6, it can be seen that H_2_O_2_ alone oxidizes TMB but produces less TMB oxide compared to the solution with the addition of AuNPs. Additionally, the presence of SQX or the aptamer alone does not affect the reaction between TMB and H_2_O_2_ (curves 4, 5, and 6).

### 3.3. Optimization of the Aptasensor

**Figure 3:**
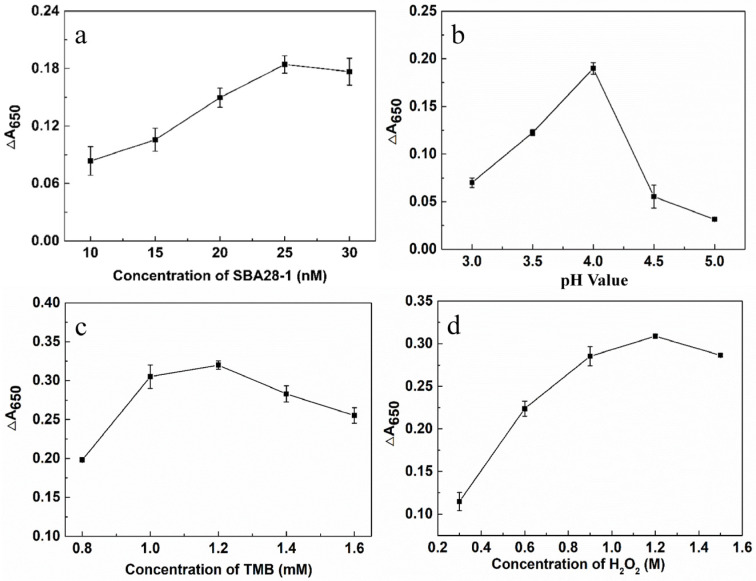
Optimization of the reaction system of the aptasensor. The change in absorbance difference (∆A_650_) of aptamer (**a**), pH value (**b**), TMB (**c**), and H_2_O_2_ (**d**) with different concentrations at 650 nm.

In order to optimize the detection conditions of the aptasensor, we evaluated the concentration of the aptamer and the pH value of the solution as well as the concentrations of TMB and H_2_O_2_. The concentration of the aptamer, as the recognition element of the target SQX, had a large impact on the sensitivity of the aptasensor. With the increase in the concentration of the aptamer, the ΔA_650_ continued to rise until the concentration reached 25 nM, at which time the ΔA_650_ peaked, indicating that all AuNPs had been encapsulated by the aptamer and its catalytic effect was optimal. Therefore, the optimal aptamer concentration for this aptasensor was determined to be 25 nM (Figure 3a). In addition, the pH of the solution affects the decomposition rate of H_2_O_2_ catalyzed by AuNPs, and a pH that is either too high or too low can lead to a decrease in the catalytic activity of the enzyme. The maximum difference in absorbance of the solution at 650 nM was observed when the pH of the solution was 4.0 (Figure 3b). Therefore, the pH of the solution was determined to be 4.0. The ∆A_650_ then reached a maximum at 1.2 mM TMB (Figure 3c), which was considered to be the optimal TMB concentration. The ΔA_650_ continued to increase until the H_2_O_2_ concentration reached 1.2 M (Figure 3d), so 1.2 M H_2_O_2_ was selected for subsequent experiments.

### 3.4. Sensitivity and Specificity of the Aptasensor

**Figure 4:**
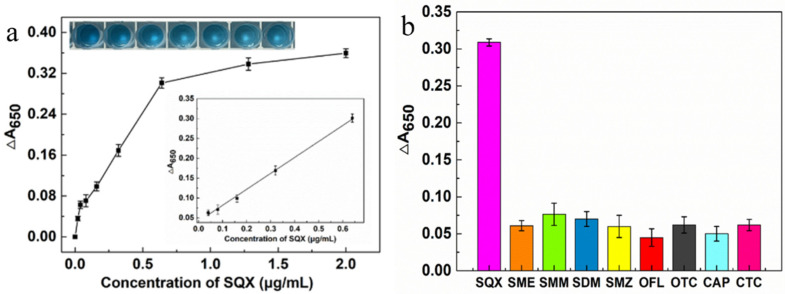
Analytical performance of the aptasensor (**a**) The linear relationship between the concentrations of SQX and the values of ∆A_650_. (**b**) The ∆A_650_ values of different kinds of drugs.

As shown in Figure 4a, a linear regression equation was obtained based on the measured absorbance values of different SQX concentrations (20, 40, 80, 160, 320, 640, 1280, and 2000 ng/mL) at 650 nm and their differences from the control. In the concentration range of 40–640 ng/mL, the absorbance difference exhibited a positive correlation with SQX concentration, resulting in the linear fitting equation Y = 0.4059X + 0.0397 (R^2^ = 0.997) and a limit of detection (LOD) value of 36.95 ng/mL. To investigate the specificity of this aptasensor, four sulfonamides (SME, SMM, SDM, and SMZ) and four non-sulfonamides (OTC, CTC, OFL, and CAP) were used to test its specificity. As shown in the Figure 4b, the ∆A_650_ values of the eight non-SQX drugs were all below 0.07, which were significantly lower than the ∆A650 values for SQX, indicating that the aptasensor had a high specificity for SQX. According to Chen et al. [39], the aptamer SBA28-1 bases A-5, G-6, and G-7 form four hydrogen bonds with SQX, the aptamer SBA28-1 bases A-5 and G-6 have three π-sulfur interactions with SQX, and the aptamer SBA28-1 base G-6 has two π-π T-shaped interactions with SQX. Such a special binding mechanism may be an important reason for the specificity exhibited by this aptasensor.

### 3.5. Validation of the Aptasensor

To evaluate the performance of the aptasensor in different water samples, various concentrations of SQX (50, 100, and 150 ng/mL) were added to SQX-free lake water and tap water. The experiment was repeated five times (n = 5) for each concentration, while the same samples were analyzed by HPLC to compare the results of both. As shown in Table 1, the recoveries of the aptasensor for the detection of SQX in lake and tap water ranged from 90.0% to 109.9%, with coefficients of variation of 4.5% to 12.6%. The recoveries ranged from 99.0% to 101.8%, with coefficients of variation of 0.9% to 3.4% when the samples were detected by HPLC. It can be seen that the present aptasensor shows a good correlation with the HPLC results. In addition, compared with other analytical methods for SQX detection (Table 2), the aptasensor constructed in this study is cheaper and simpler to fabricate and has a higher reliability, making it promising for detecting SQX in various water samples.

## 4. Conclusions

Here, we successfully constructed an aptasensor for the detection of SQX utilizing the principle that AuNPs catalyze the oxidation of TMB by H_2_O_2_, while the aptamer enhances the catalytic effect of the AuNPs. Compared with the traditional instrumental methods, the aptasensor is simpler and more convenient, which is more suitable for rapid detection on site. Unlike immunological methods that require specific antibodies, the aptamer, as a novel recognition molecule, is more readily available than antibodies, and the aptasensor is also able to rapidly detect SQX residues. In addition, avoiding the use of expensive labels such as fluorescent dyes or nanoparticles to modify the aptamer, the aptasensor becomes more cost-effective, easier to prepare, and widely available. The aptasensor constructed in this paper demonstrated good linearity (Y = 0.4059X + 0.0397, R^2^ = 0.997) at SQX concentrations ranging from 40 to 640 ng/mL, with the lowest detection limit being 36.95 ng/mL. The spiked recoveries of lake water and tap water ranged from 90% to 109.9%, with coefficients of variation from 4.5% to 12.6%. The detection results of the aptasensor and HPLC showed a good correlation. The results showed that the aptasensor can effectively detect residual SQX in aqueous environments, and its detection range is wide, which has good prospects for other applications.

## Figures and Tables

**Table 1:** Mean recoveries and coefficient of variation for the SQX in the spiked samples using aptasensor and HPLC (n = 5).

Sample	Spiked (µg/kg)	Aptasensor		HPLC	
		Recovery (%) ± SD	CV (%)	Recovery (%) ± SD	CV (%)
Lake Water	50.0	93.0 ± 4.2	4.5	99.0 ± 1.9	2.0
	100.0	97.8 ± 10.4	10.6	99.3 ± 1.1	1.1
	150.0	109.9 ± 9.5	8.6	101.8 ± 3.4	3.4
Tap Water	50.0	90.0 ± 6.8	7.5	99.3 ± 1.0	1.0
	100.0	101.2 ± 9.2	9.1	101.1 ± 0.9	0.9
	150.0	106.3 ± 13.4	12.6	101.6 ± 2.9	2.9

SD: standard deviation; CV: coefficient of variation.

**Table 2:** Comparison of the performance of different detection methods for the detection of SQX.

Method	Sample	Range	LOD	References
HPLC	River water	98.5–2756.5 ng/mL	60.5 ng/mL	[9]
	Chicken, Pork, Egg	0.05–10.0 ng/mL	0.01 ng/mL	[10]
	A.S.K Powder	12–26 μg/mL	-	[11]
LC-MS/MS	Water matrices	-	3.0 ng/L	[12]
UHPLC-MS/MS	Fish, Shrimp, Crab	1.0–50 ng/L	7.04 ng/kg	[13]
	Clay and Sand	0.5–1.5 ng/g	-	[14]
CE	Sea water	1.0–100 ng/mL	0.15 ng/mL	[15]
	Ophthalmic solution	50–250 μg/mL	17 μg/mL	[16]
ICA	Egg, Chicken Muscle	0.01–100 ng/mL	-	[17]
ELISA	Chicken tissues	2.5–60 ng/mL	2.5 ng/mL	[45]
Immunoassay	Milk	0.1–1000 ng/mL	2.95 ng/mL	[46]
Fluorescence immunoassay	Milk, Chicken, Shrimp	0.1–100 ng/mL	0.1 ng/mL	[47]
	Pork, Chicken, Fish	0.01–100 ng/mL	0.04 ng/mL	[48]
Carbon paste sensor	Blood serum, Urine, Milk	5.0–10,000 μM	3.0 μM	[49]
Fluorescent aptasensor	Fish	1.0–10.0 μM	0.20 μM	[29]
	Milk	0.05–50 ng/mL	0.11 ng/mL	[38]
Electrochemical aptasensor	Pork	1 pg/mL–100 ng/mL	0.547 pg/mL	[30]
Colorimetric aptasensor	Lake Water, Tap water	40–640 ng/mL	36.95 ng/mL	This work

-: not mentioned.

## Data Availability

Data are contained within the article.
